# Diseases of Infants and Children

**Published:** 1850-05

**Authors:** 


### ARTICLE III.

*On the Diseases of Infants and Children.*—By Fleetwood Churchill, M.D., M.R.I.A.; Hon. Fellow of the College of Physicians, Ireland; Hon. Member of the Philadelphia Medical Society, etc., etc.; Author of "The Theory and Practice of Midwifery," "on the Diseases of Females," etc. pp. 623. oct: Philadelphia: Lea & Blanchard: 1850.—(From the Publishers, by Express.)

We have not time to read and give an extended notice of this work in the present number. The writings of the author are familiar to the profession in America; and from the high reputation of former works, we doubt not this will receive an extensive patronage.

One new feature presented, we must notice, i.e., it was written expressly for the American Publishers. This is then not a re-print of a European work, but an Original European work published in America. While we award to Dr. Churchill great ability in compiling medical works, we very much doubt whether he will have furnished a work better adapted to the wants of American Practitioners, than those of some of our native authors upon the same subject. While the enterprising publishers are certainly not to blame for publishing such works as sell best, it is humiliating to our national pride to see with what avidity every thing in the way of medical literature from Europe, is gathered up by the profession, and often to the great neglect of equally good, if not better native productions.

We will endeavor to give a more extended notice of this work heieafter. E.

